# Integrated network toxicology and multi-omics profiling reveal cell cycle, DNA damage repair, and metabolic alterations in cyclophosphamide-induced premature ovarian insufficiency

**DOI:** 10.3389/fphar.2026.1857647

**Published:** 2026-09-24

**Authors:** Zhaoyang Yu, Lidan Liu, Xiqian Zhang, Ying Li, Yingqi Nong, Lingyu Ke, Mujun Li, Yuxia Wang, Huimei Wu, Fenghua Liu

**Affiliations:** 1 Reproductive Medicine Center, The First Affiliated Hospital of Jinan University, Guangzhou, China; 2 Reproductive Medicine Center, Guangdong Women and Children Hospital, Guangzhou, China; 3 Reproductive Medicine Center, The First Affiliated Hospital of Guangxi Medical University, Nanning, China; 4 School of Pharmaceutical Sciences, Guangzhou Medical University, Guangzhou, China

**Keywords:** premature ovarian insufficiency, cyclophosphamide, network toxicology, reproductive toxicity, multi-omics, in silico perturbation

## Abstract

**Background:**

Cyclophosphamide (CTX)-induced chemotherapy injury is a leading iatrogenic cause of premature ovarian insufficiency (POI) and a major barrier to fertility preservation. Yet the systems-level mechanisms underlying its reproductive toxicity remain poorly defined.

**Methods:**

An integrated network toxicology framework incorporating machine learning and Mendelian randomization (MR) was applied to identify candidate targets, evaluate genetic associations, and establish a predictive model. Ovarian transcriptomic and metabolomic profiling, together with *in vivo* validation, were subsequently performed in a CTX-induced POI rat model. Single-cell-based *in silico* perturbation analysis was further conducted to investigate the potential cellular functions and mechanisms of key candidate targets in POI.

**Results:**

Integrated bioinformatic analyses and machine learning prioritized six candidate targets associated with CTX-induced ovarian injury, with PLS regression consistently identified as the optimal predictive model. SHAP analysis revealed CXCL1 and COL4A4 as the major contributors to model prediction. MR supported genetic associations between genetically predicted CXCL1/COL4A4 expression and POI risk. Immunohistochemical validation confirmed protein expression patterns consistent with transcriptomic findings. Multi-omics profiling revealed a convergent ovarian injury signature characterized by impaired cell-cycle regulation (G2/M checkpoint), defective DNA damage repair and mitotic function (mitotic spindle), enhanced apoptotic signaling, and coordinated suppression of folate metabolism, ubiquinone biosynthesis, and cytochrome P450-mediated drug metabolism pathways. Single-cell-based *in silico* perturbation analysis further suggested that CXCL1 and COL4A4 may contribute to ovarian dysfunction through regulation of inflammatory responses and extracellular matrix remodeling.

**Conclusion:**

This study shows that CTX-induced ovarian injury is accompanied by coordinated alterations in immune-inflammatory signaling, cell-cycle and DNA damage responses, and metabolic homeostasis. CXCL1 and COL4A4 were prioritized as candidate biomarkers associated with POI risk, providing potential targets for further mechanistic investigation and fertility-preserving interventions.

## Graphical Abstract

**Figure:**
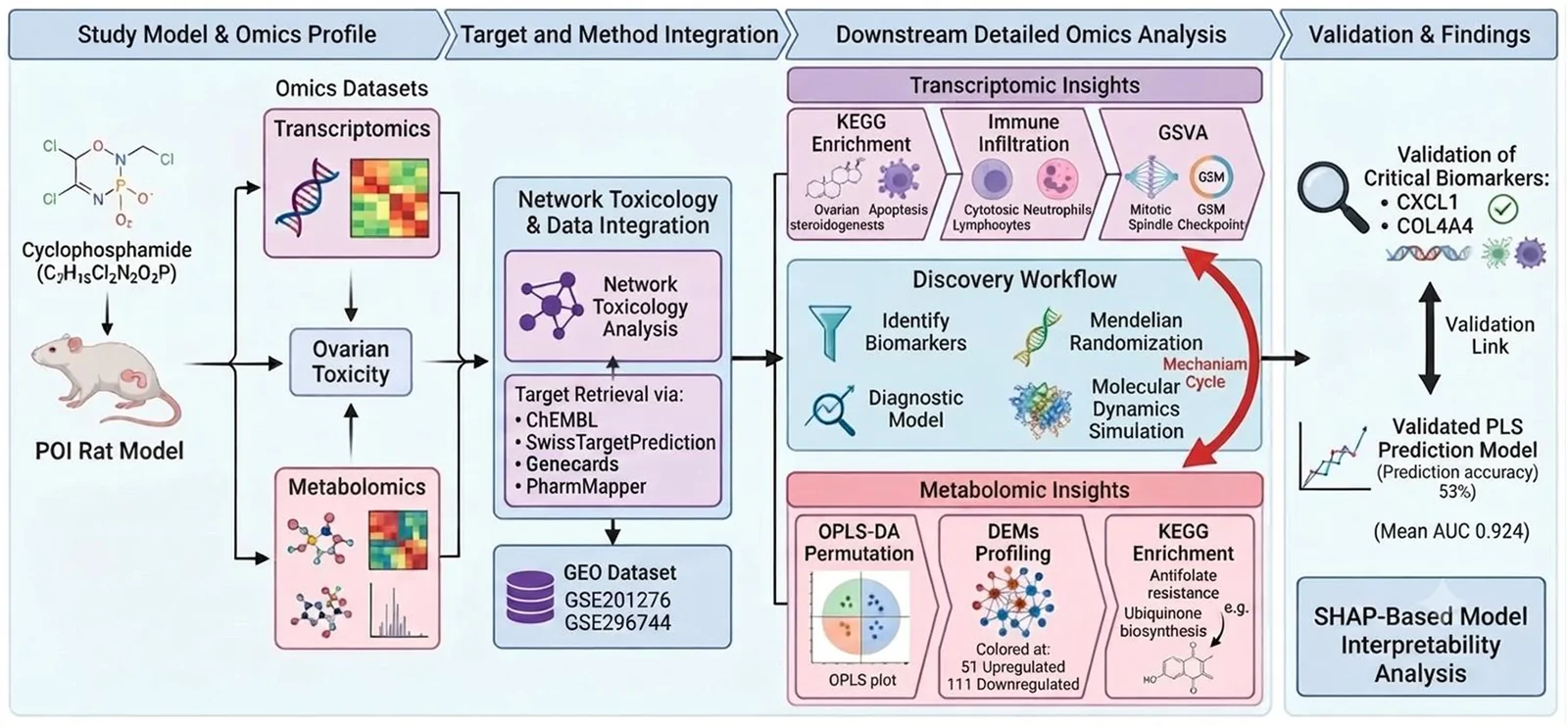

## 1 Introduction

Premature ovarian insufficiency (POI) is a reproductive endocrine disorder characterized by follicular depletion, elevated gonadotropins, and hypoestrogenism, clinically presenting with menstrual disturbance, infertility, and perimenopausal symptoms (Guo et al., 2025). Sustained estrogen deficiency substantially increases the risk of long-term comorbidities, including cardiovascular disease, osteoporosis, and neurodegenerative disease (Panay et al., 2024). POI is etiologically heterogeneous, among which chemotherapy-related ovarian injury represents one of the most common and clinically relevant iatrogenic causes. Chemotherapeutic agents can trigger follicular loss and granulosa-cell apoptosis and disrupt ovarian microenvironmental homeostasis, thereby impairing intercellular metabolic support and signaling crosstalk and accelerating ovarian functional decline (Guo et al., 2024). With improved cancer survival and an expanding population of reproductive-age female survivors, treatment-induced ovarian damage has become a major barrier to fertility preservation and long-term reproductive health. Therefore, identifying actionable mechanisms and protective targets that mitigate chemotherapy-associated ovarian injury without compromising anticancer efficacy remains a shared clinical and translational challenge at the interface of oncology and reproductive medicine.

Cyclophosphamide (CTX) is a classical alkylating agent extensively used in the treatment of malignant tumors and autoimmune diseases (Gonçalves et al., 2009). Accumulating evidence indicates that CTX has adverse effects on the reproduction of both females and males (Fang et al., 2022; Jiang et al., 2020). However, the underlying mechanism of its systemic reproductive toxicity has not yet been fully elucidated. CTX can promote premature ovarian insufficiency by accelerating aberrant activation of primordial follicles and inducing atresia of growing follicles, ultimately leading to early depletion of the follicular reserve (Spears et al., 2019). Among patients with systemic lupus erythematosus receiving long-term moderate-dose CTX therapy, the risk of persistent amenorrhea exhibits a strong age dependency, affecting approximately 12% of women younger than 25 years and more than 50% of those older than 30 years (Emadi et al., 2009). At the mechanistic level, CTX is metabolized in the liver into the highly reactive intermediate 4-hydroxycyclophosphamide, which induces DNA crosslinking and double-strand breaks, enhances oxidative stress, disrupts mitochondrial membrane potential, and promotes lipid peroxidation within ovarian tissue (Pass et al., 2005). Collectively, CTX serves as a key and highly clinically relevant model drug for elucidating the mechanisms of chemotherapy-induced ovarian damage and for identifying potential fertility-protective therapeutic targets.

POI is an outcome of chemotherapy-induced ovarian injury and is also regarded as a comprehensive risk model, reflecting the high sensitivity of the female reproductive system to chemical stressors. However, existing studies on chemotherapy-associated ovarian damage have largely focused on individual molecules or isolated signaling pathways, leaving a lack of systems-level toxicological frameworks. Network toxicology enables a systematic dissection of chemical-induced toxicity by capturing complex multi-target interactions and pathway perturbations (Yan et al., 2026). Multi-omics approaches further enhance mechanistic inference by providing multidimensional views of biological activities within complex systems (Zhu et al., 2022). Accordingly, CTX was selected as a model compound, and an integrative strategy combining network toxicology, machine learning (ML), MR was employed to prioritize toxicity-related targets and develop predictive models. In parallel, transcriptomic and metabolomic profiling of ovaries from a POI rat model was performed to characterize CTX-induced gene and metabolic alterations and the associated regulatory pathways, followed by *in vivo* validation of key targets. Finally, the potential cellular functions and mechanisms of key candidate targets in POI were explored through single-cell *in silico* perturbation analysis. Overall, this study aims to construct a systems-level toxicological network of CTX-induced chemotherapy-associated ovarian injury, providing a mechanistic basis and actionable targets for fertility preservation and targeted intervention strategies.

## 2 Methods

### 2.1 Dataset acquisition and preprocessing

The POI-related transcriptomic datasets GSE201276 and GSE296744 were retrieved from the Gene Expression Omnibus (GEO, https://www.ncbi.nlm.nih.gov/geo/). Probe identifiers in the SOFT formatted family files were converted to official gene symbols, and expression values were log2-transformed for normalization. The two datasets were then merged, and batch effects were corrected using the limma package. Principal component analysis (PCA) before and after correction was visualized using the ggplot2 package to assess the effectiveness of batch adjustment. Differentially expressed genes (DEGs) associated with POI were identified using the limma package, with significance thresholds set at *P* < 0.05 and |log fold change (FC)| > 0.5.

### 2.2 Identification of CTX-associated genes

CTX-associated genes were compiled from four complementary databases as an initial hypothesis-generating set. “Cyclophosphamide” was queried in ChEMBL (https://www.ebi.ac.uk/chembl/) and GeneCards (https://www.genecards.org/) to retrieve experimentally annotated compound–protein associations and broader drug-responsive or literature-associated genes, respectively. The CTX structure and SMILES notation were obtained from PubChem (https://pubchem.ncbi.nlm.nih.gov/) and submitted to PharmMapper (https://www.lilab-ecust.cn/pharmmapper/) for pharmacophore-based target prediction and SwissTargetPrediction (http://www.swisstargetprediction.ch/) for ligand similarity-based prediction. Because these resources represent different levels of evidence, the retrieved genes were regarded as putative CTX-associated genes rather than confirmed direct targets. All entries were standardized, merged, and deduplicated before downstream disease-context and data-driven filtering.

### 2.3 Weighted gene co-expression network analysis (WGCNA)

The integrated GEO dataset was used for WGCNA construction and machine-learning model development. WGCNA was conducted to identify co-expression modules associated with the POI phenotype. After preprocessing the expression matrix and removing low-expression genes, the top 10,000 most variable genes—ranked by median absolute deviation—were selected for network construction. An appropriate soft-thresholding power was determined using the pickSoft threshold function to approximate scale-free topology. The resulting adjacency matrix was transformed into a topological overlap matrix (TOM), followed by hierarchical clustering and module detection using the dynamic tree-cut algorithm. Module–trait correlations were assessed using Pearson correlation analysis, and modules significantly associated with POI (|R| > 0.3, *P* < 0.05) were identified as key modules for downstream analysis.

### 2.4 Identification of key targets of cyclophosphamide in POI

Key genes mediating the effects of CTX in POI were identified by intersecting three gene sets: CTX-related targets, POI-associated differentially expressed genes, and hub genes from WGCNA modules. To further refine the candidate set, least absolute shrinkage and selection operator (LASSO) regression was performed using the glmnet package. Ten-fold cross-validation was applied to determine the optimal regularization parameter (λ) that minimized prediction error. Genes with non-zero coefficients in the final model were retained as candidate key targets, as their coefficients represent the strength and direction of association with the POI phenotype. Coefficient profiles were visualized using two-dimensional trajectory plots to illustrate the shrinkage path and feature selection process.

### 2.5 GO and KEGG enrichment analysis

Gene Ontology (GO) and Kyoto Encyclopedia of Genes and Genomes (KEGG) enrichment analyses were performed using the clusterProfiler R package to elucidate the biological functions and signaling pathways associated with the intersection genes. GO enrichment included the three major ontologies—biological process (BP), cellular component (CC), and molecular function (MF). Terms and pathways with *P* < 0.05 were considered significantly enriched. For both GO and KEGG analyses, the top 10 enriched categories were selected based on adjusted significance and enrichment scores. The enrichment results were visualized as bar plots to provide an intuitive representation of the most prominent functional categories and pathways.

### 2.6 Establishment and evaluation of the POI rat model

Female Sprague–Dawley rats (200–220 g) were obtained from the Experimental Animal Center of Guangxi Medical University and maintained under specific pathogen-free conditions with free access to food and water. After a 1-week acclimation period, animals were randomly assigned to the control group or CTX-induced POI group (n = 6 per group). POI was induced by intraperitoneal administration of cyclophosphamide (60 mg/kg on day 1 followed by 8 mg/kg/day from days 2–14), whereas control animals received equivalent volumes of physiological saline. At the end of the induction period, rats were anesthetized with isoflurane, and bilateral ovaries and peripheral blood samples were collected. One ovary from each animal was used for transcriptomic analysis, while the contralateral ovary was subjected to untargeted metabolomic analysis. Histological evaluation was performed using hematoxylin and eosin (HE) staining, and follicle numbers at different developmental stages were quantified by blinded observers. Serum AMH, FSH, and E2 levels were measured using ELISA. All animal procedures were approved by the Animal Ethics Committee of Guangxi Medical University (Approval No. 20250531).

### 2.7 Full-length transcriptome sequencing and bioinformatics analysis

Total RNA was extracted from rat ovarian tissues, quality-controlled, and used for cDNA library construction. Full-length transcriptome sequencing was performed on the Oxford Nanopore Technologies (ONT) platform to obtain high-contiguity transcripts. Raw reads were processed through standard ONT workflows for filtering, alignment, and quantification, and gene expression levels were normalized using counts per million (CPM). Differentially expressed genes (DEGs) were identified based on | Log FC| > 0.5 and *P* < 0.05, and results were visualized using volcano plots, MA plots, and hierarchical clustering heatmaps. Functional annotation and enrichment analyses of DEGs were conducted using GO and KEGG database. Protein–protein interaction (PPI) networks were constructed using the STRING database to identify potential regulatory hub genes. The ONT transcriptome dataset was further used as an independent validation cohort to assess the robustness and potential translational applicability of the CTX-related POI target gene model. Diagnostic performance of individual key targets in the POI rat model was evaluated by calculating the area under the ROC curve (AUC).

### 2.8 Immunohistochemistry analysis

Ovarian tissues were fixed, paraffin embedded, and sectioned for immunohistochemical (IHC) analysis. After deparaffinization and heat-induced antigen retrieval, sections were blocked with serum to minimize nonspecific staining and incubated with primary antibodies against CXCL1 (ElabSCIENCE, China; dilution 1:100) or COL4A4 (Abmart, China; dilution 1:100) overnight at 4 °C. Following incubation with HRP-labeled secondary antibodies, antigen signals were developed using DAB substrate and counterstained with hematoxylin. Representative images were captured using a microscope, and the relative expression levels were quantified based on staining intensity and positive staining area.

### 2.9 GSVA pathway scoring

Gene Set Variation Analysis (GSVA) was performed on the ovarian transcriptomic dataset using the HALLMARK gene sets to calculate sample-wise pathway activity scores. Pathway scores were compared between the POI and control groups to identify functional alterations associated with CTX-induced ovarian injury. Pearson correlation analysis was subsequently conducted to assess the associations between the five model-derived marker genes and pathway activity. A *P* value <0.05 was considered statistically significant.

### 2.10 Untargeted metabolomics (LC–QTOF) and multi-omics integration analysis

Metabolites were extracted from ovarian tissues collected from CTX-induced POI rats and control animals (n = 6 per group) and analyzed using an untargeted LC–QTOF metabolomics platform comprising a Waters Acquity I-Class PLUS UPLC system coupled with a Waters Xevo G2-XS QTOF mass spectrometer. Raw data were processed through peak extraction, alignment, quantification, and batch correction to generate a high-quality metabolite matrix. Principal component analysis (PCA) was performed to evaluate overall metabolic separation and within-group variation, while orthogonal partial least-squares discriminant analysis (OPLS-DA) was applied to identify discriminative metabolic features. Differential metabolites were screened using an integrated criterion combining VIP >1, absolute fold change >2, and nominal *P* < 0.05. These metabolites were subsequently subjected to pathway enrichment analysis to characterize CTX-associated metabolic alterations.

### 2.11 Construction and validation of diagnostic models using multiple machine learning algorithms

To identify the optimal diagnostic model, a panel of 17 machine learning algorithms was applied to the previously selected target genes, including random forest (rf), extreme gradient boosting (xgbTree), radial basis SVM (svmRadial), generalized linear model (glm), k-nearest neighbors (knn), partial least squares (pls), neural network (nnet), naïve Bayes (nb), linear discriminant analysis (lda), weighted k-nearest neighbors (kknn), stepwise LDA (stepLDA), weighted radial SVM (svmRadialWeights), stepwise QDA (stepQDA), linear SVM (svmLinear), LogitBoost, generalized partial least squares (plsRglm), and stepwise GLM (glmStepAIC). To minimize variability caused by dataset partitioning, the merged GEO dataset was used as the training set, whereas transcriptomic data from the CTX-induced POI rat model served as the external validation set. Model performance was primarily evaluated using the area under the ROC curve (AUC), which allowed direct comparison across algorithms to determine the highest-performing classifier.

### 2.12 Comprehensive performance assessment and SHAP-based interpretability analysis

To comprehensively assess the robustness and clinical applicability of the diagnostic models, several evaluation approaches were implemented. Calibration curves were plotted to examine the agreement between predicted probabilities and observed outcomes. Decision curve analysis (DCA) was applied to quantify the net clinical benefit across a range of threshold probabilities. Precision–recall (P–R) curves were generated to further evaluate model performance under class-imbalanced conditions. To enhance interpretability, Shapley Additive Explanations analysis (SHAP) was conducted to quantify the contribution of each feature gene to model predictions. SHAP visualization plots clearly illustrated the direction and magnitude of individual features’ effects, providing biologically meaningful insights into the model’s decision-making process.

### 2.13 Construction of the TF–miRNA–mRNA regulatory network

MicroRNAs (miRNAs) targeting the five key genes were predicted using the ENCORI database (https://rnasysu.com/encori/). Transcription factors (TFs) regulating these genes were identified from the KnockTF database (http://www.licpathway.net/KnockTF/index.php). The integrated regulatory network comprising target genes, TFs, and miRNAs was visualized using Cytoscape software to elucidate the multi-layered post-transcriptional and transcriptional regulatory interactions.

### 2.14 Mendelian randomization (MR) analysis

Cis-eQTL summary statistics were obtained from the eQTLGen consortium, and SNPs associated with each feature gene were extracted as exposure variables. GWAS summary data for POI (finngen_R10_E4_OVARFAIL) were retrieved from the FinnGen study and used as the outcome dataset. Strong instrument variables were selected as SNPs significantly associated with the exposure (P < 4 × 10−8), and linkage disequilibrium was removed using the clump_data function. Multiple MR approaches—including inverse variance weighted (IVW), maximum likelihood, MR-Egger, weighted median, and weighted mode—were applied to estimate the causal effects of feature genes on POI. Horizontal pleiotropy was assessed using the MR-Egger intercept test, and heterogeneity across SNP-specific Wald ratios was evaluated using Cochran’s Q statistic under both IVW and MR-Egger models.

### 2.15 Single-cell RNA sequencing data processing and cell annotation

Single-cell RNA sequencing data from the ovarian tissue dataset GSE334364 were analyzed using the Seurat package in R. Low-quality cells were removed based on the criteria of 500 < *nFeature_RNA* < 6000 and mitochondrial gene proportion (*percent.mt*) < 20%. Following normalization and dimensionality reduction, unsupervised clustering was performed using the *FindClusters* function. Cell populations were annotated according to the expression patterns of established marker genes, including granulosa cells, macrophages, endothelial cells, stromal cells, epithelial cells, luteal cells, and theca cells.

### 2.16 Single-cell-based *in silico* perturbation analysis

To investigate the potential functional effects of *Cxcl1* and *Col4a4* perturbation in ovarian cell populations, a single-cell transcriptome-based virtual knockout analysis was performed using the scTenifoldKnk. Gene regulatory networks were inferred from single-cell expression profiles using repeated subsampling and network reconstruction, followed by tensor-based integration to generate consensus regulatory networks. Virtual perturbation was simulated by removing the outgoing regulatory effects of the target genes from the reconstructed networks. The downstream regulatory alterations were quantified by comparing the perturbed and original network states, and significantly affected genes were subsequently subjected to functional enrichment analysis.

## 3 Results

### 3.1 Identification of POI-Related differential genes and CTX-associated targets

After merging the two transcriptomic datasets from GEO, normalization and batch-effect correction were performed, followed by PCA to assess the correction efficiency. The PCA plot showed that samples from the two datasets were well mixed and distributed across the first two principal components after correction, indicating successful removal of batch effects (Figure 1A). Using *P* < 0.05 and |logFC| > 0.5 as the selection criteria, a total of 844 POI-related DEGs were identified, including 330 upregulated and 514 downregulated genes (Figures 1B,C, Supplementary data-1). CTX–associated targets were systematically collected from four major databases. Specifically, 121, 196, 98, and 2,443 potential targets were retrieved from ChEMBL, PharmMapper, SwissTargetPrediction, and GeneCards, respectively (Supplementary data-2). After merging and removing duplicates, 2,657 CTX-associated genes were retained as an initial candidate pool. These genes represented heterogeneous evidence, ranging from predicted compound–protein associations to drug-responsive and literature-associated genes, and were therefore subjected to subsequent disease-context and data-driven filtering.

**FIGURE 1:**
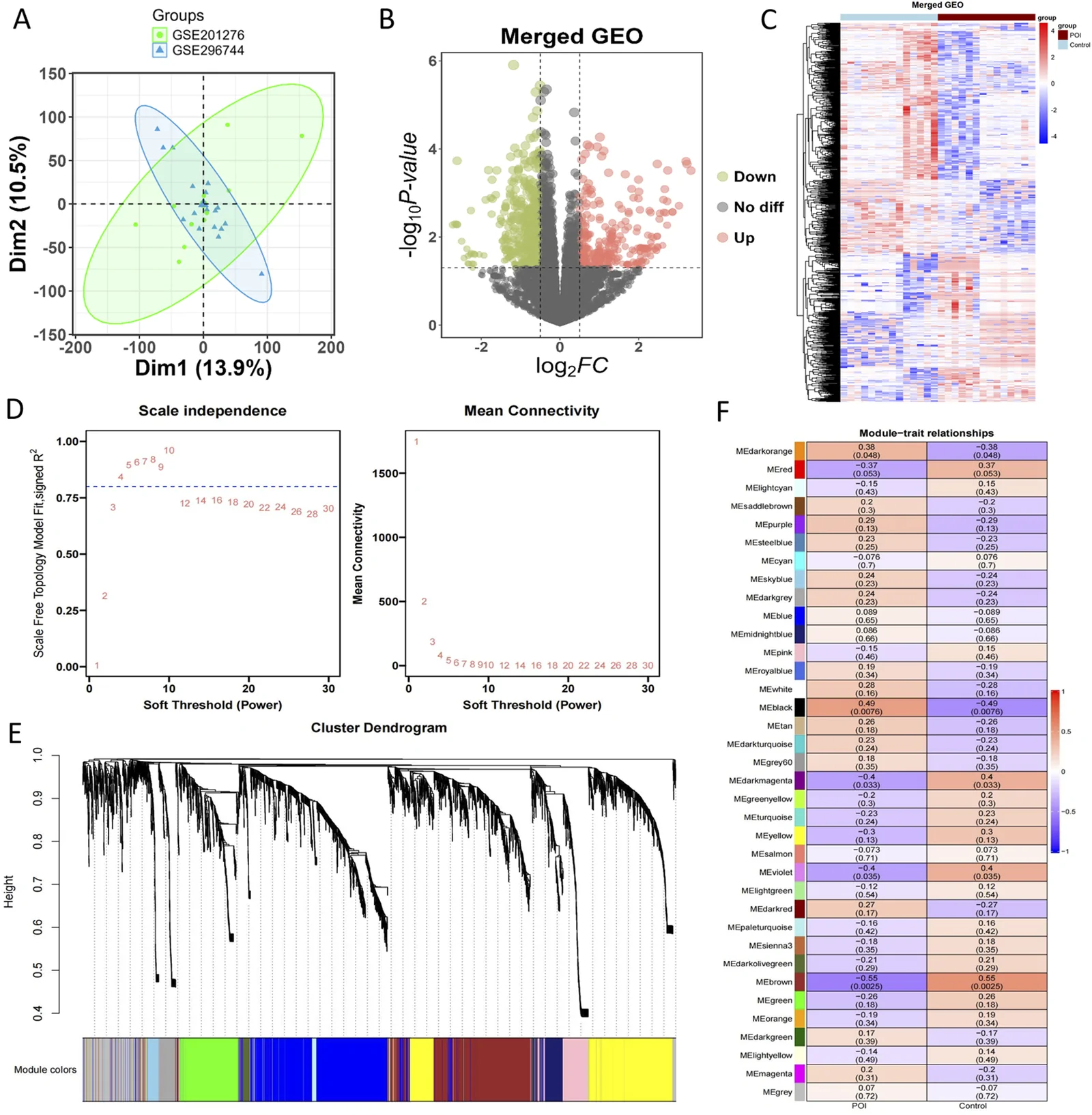
Identification of key gene modules in the GEO dataset using WGCNA. **(A)** Principal component analysis (PCA) plot of the merged GEO datasets after batch effect correction. **(B)** Volcano plot showing DEGs in the GEO dataset. **(C)** Cluster heatmap of the merged GEO dataset. **(D)** Scale-free topology analysis plot. **(E)** Hierarchical clustering dendrogram of genes and module identification based on topological overlap. **(F)** Module–trait relationship analysis showing correlations between co-expression modules and clinical traits.

### 3.2 Identification of key modules and hub genes via WGCNA

WGCNA identified the optimal soft-thresholding power, with a power of four yielding a scale-free topology fit index of R^2^ > 0.8 (Figure 1D). A total of 36 co-expression modules were detected, and their clustering relationships were visualized using dynamic tree cutting and heatmaps, with the grey module representing unassigned genes (Figure 1E). Based on the correlations between module eigengenes and phenotype (|R| > 0.3, *P* < 0.05), five key modules were selected: MEbrown, MEblack, MEdarkmagenta, MEviolet, and MEdarkorange. Using intramodular connectivity (kME >0.3) as an additional criterion, 1,115 hub genes were identified within these key modules (Figure 1F). Intersection analysis of DEGs, WGCNA-derived hub genes, and CTX-associated targets ultimately yielded 50 candidate genes implicated in CTX-induced ovarian injury (Figures 2A,B).

**FIGURE 2:**
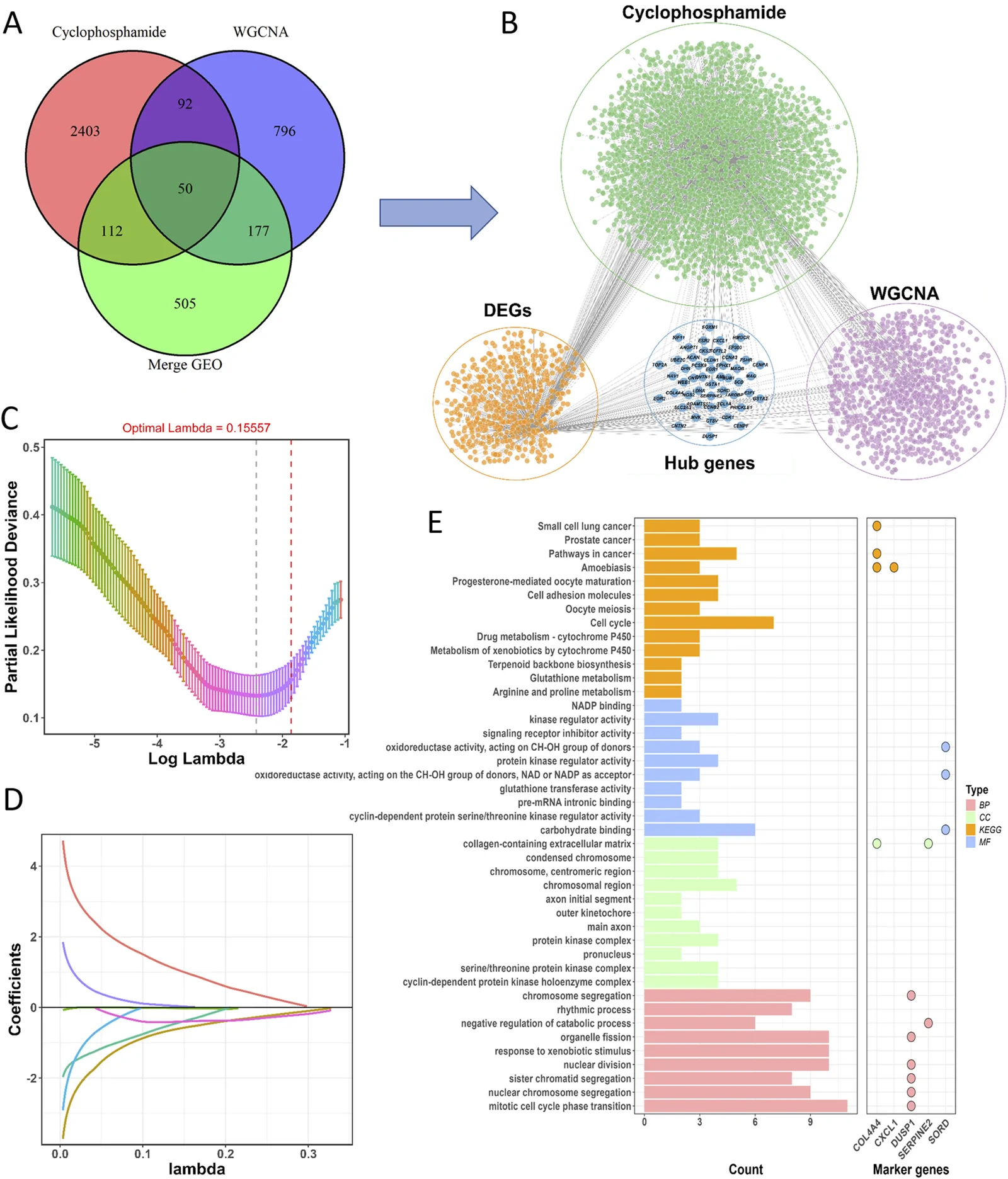
Identification of CTX-associated targets and functional enrichment analysis. **(A,B)** Venn diagram and interaction network of overlapping genes among CTX-related targets, DEGs, and core gene from WGCNA modules. **(C,D)** LASSO regression coefficient profiles and cross-validation error curves for feature selection. **(E)** KEGG pathway enrichment analysis of DEGs.

### 3.3 GO and KEGG enrichment analysis of the intersection genes

KEGG enrichment analysis shows that the intersection genes are mainly enriched in pathways closely associated with ovarian function and metabolic regulation, including progesterone-mediated oocyte maturation, cell adhesion molecules, oocyte meiosis, cell cycle, glutathione metabolism, and arginine and proline metabolism, suggesting intersection genes may involve in oocyte development, cell-cycle regulation, and oxidative stress responses. GO analysis further revealed enrichment across all three major categories. In the MF category, enriched terms included kinase regulator activity, oxidoreductase activity, glutathione transferase activity, and cyclin-dependent protein serine/threonine kinase regulator activity. The CC terms were enriched in the collagen-containing extracellular matrix, condensed chromosome, chromosome centromeric region, protein kinase complex, and cyclin-dependent protein kinase holoenzyme complex. For the BP category, significant enrichment was observed in chromosome segregation, sister chromatid segregation, and mitotic cell-cycle phase transition, indicating that these genes are functionally linked to chromosomal dynamics, cell division, and ovarian microenvironment regulation (Figure 2E, Supplementary Data 3).

### 3.4 Identification of candidate biomarker genes by LASSO regression

A LASSO regression model was applied to the 50 intersection genes, with 10-fold cross-validation used to determine the optimal penalty parameter. The coefficient trajectory plot demonstrated progressive shrinkage of most gene coefficients as λ increased, with only a subset retaining non-zero coefficients (Figure 2C). The cross-validation curve identified λ = 0.15557 as the value yielding the minimum deviance (Figure 2D). At this optimal λ, six genes—SORD, COL4A4, CXCL1, DUSP1, HMGCR, and SERPINE2—remained in the model, representing robust candidate biomarkers for subsequent modelling and mechanistic analyses.

### 3.5 Multi-classification model identify the optimal diagnostic model

Integrated evaluation across 17 machine-learning classifiers showed that the average AUCs ranged from 0.657 to 0.924 (Figures 3A–C), among which the PLS model achieved the best overall performance with an AUC of 0.959 in the training set, 0.889 in the validation set, and a mean AUC of 0.924. This model further retained five key signature genes—SORD, COL4A4, CXCL1, DUSP1, and SERPINE2—as the final diagnostic panel (Figures 3E,F). Most algorithms demonstrated high specificity approaching 1.0, while models such as plsRglm, svmLinear, xgbTree, and pls exhibited the smallest variability, indicating greater stability (Figure 3D). The clinical utility and robustness of the PLS model were subsequently assessed using multiple evaluation curves. The PR curve showed consistently high precision across a wide recall range, supporting strong discriminatory power and a low false-positive rate even under class-imbalanced conditions (Figure 3G). The calibration curve demonstrated reasonable agreement between predicted and observed probabilities; although mild deviations were observed in certain intervals, the overall trend indicated satisfactory calibration and predictive reliability (Figure 3H). DCA further revealed that the PLS classifier provided a consistently higher net benefit than the “treat-all” and “treat-none” strategies within threshold probabilities of 0–0.7, highlighting its potential clinical value for assessing CTX-induced ovarian toxicity (Figure 3I). Collectively, these analyses identify the PLS model as the clinically useful diagnostic classifier.

**FIGURE 3:**
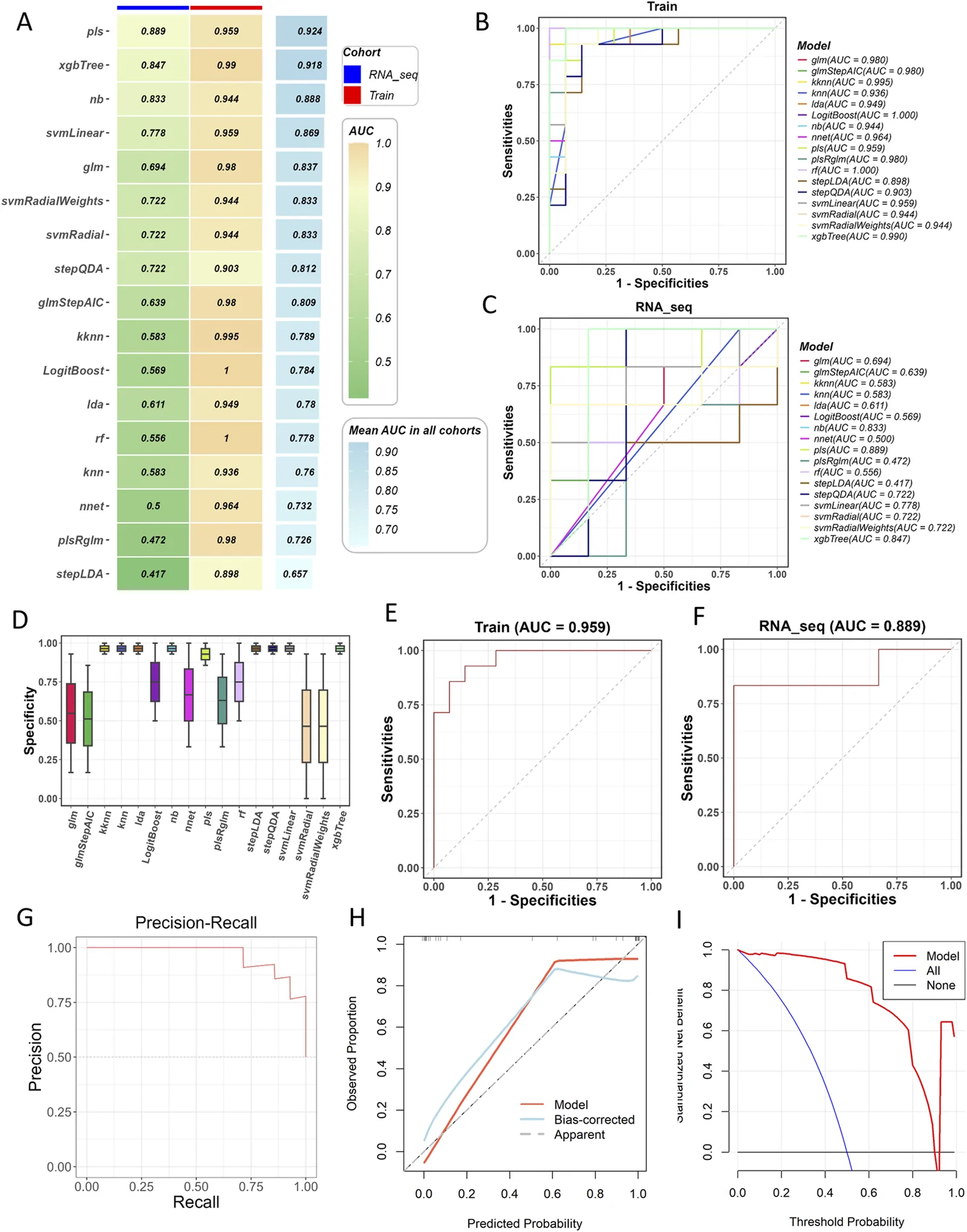
Identification of the optimal predictive model based on multiple machine learning algorithms. **(A)** Heatmap of AUC values for 17 machine learning models. **(B–D)** ROC curves in the training and validation sets and boxplots of specificity across the 17 models. **(E,F)** ROC curves of the PLS model in the training and validation sets. **(G–I)** Precision–recall curve, calibration curve, and decision curve analysis of the PLS model.

### 3.6 SHAP-based model interpretability analysis

The SHAP bar plot identified CXCL1 as the strongest contributor to model predictions, followed by COL4A4, SORD, SERPINE2, and DUSP1 (Figure 4A). In the beeswarm plot, the color gradient from purple to yellow reflects increasing gene expression levels. High-expression samples of CXCL1 and COL4A4 predominantly clustered on the positive SHAP side, indicating that elevated expression of these genes drives the model toward predicting CTX-induced ovarian injury. In contrast, high-expression SORD samples were mainly distributed on the negative SHAP side, suggesting a protective role that decreases the predicted risk. SERPINE2 and DUSP1 showed mixed distributions across positive and negative SHAP values, implying greater heterogeneity in their contributions to the model output (Figure 4B). The SHAP force plot further demonstrated the contribution of each gene in a representative sample: CXCL1 contributed the largest positive shift (+0.0849). Collectively, these genes increased the model’s predicted probability from the baseline value (E [f(x)] = 0) to a final output of 0.191 (Figure 4C).

**FIGURE 4:**
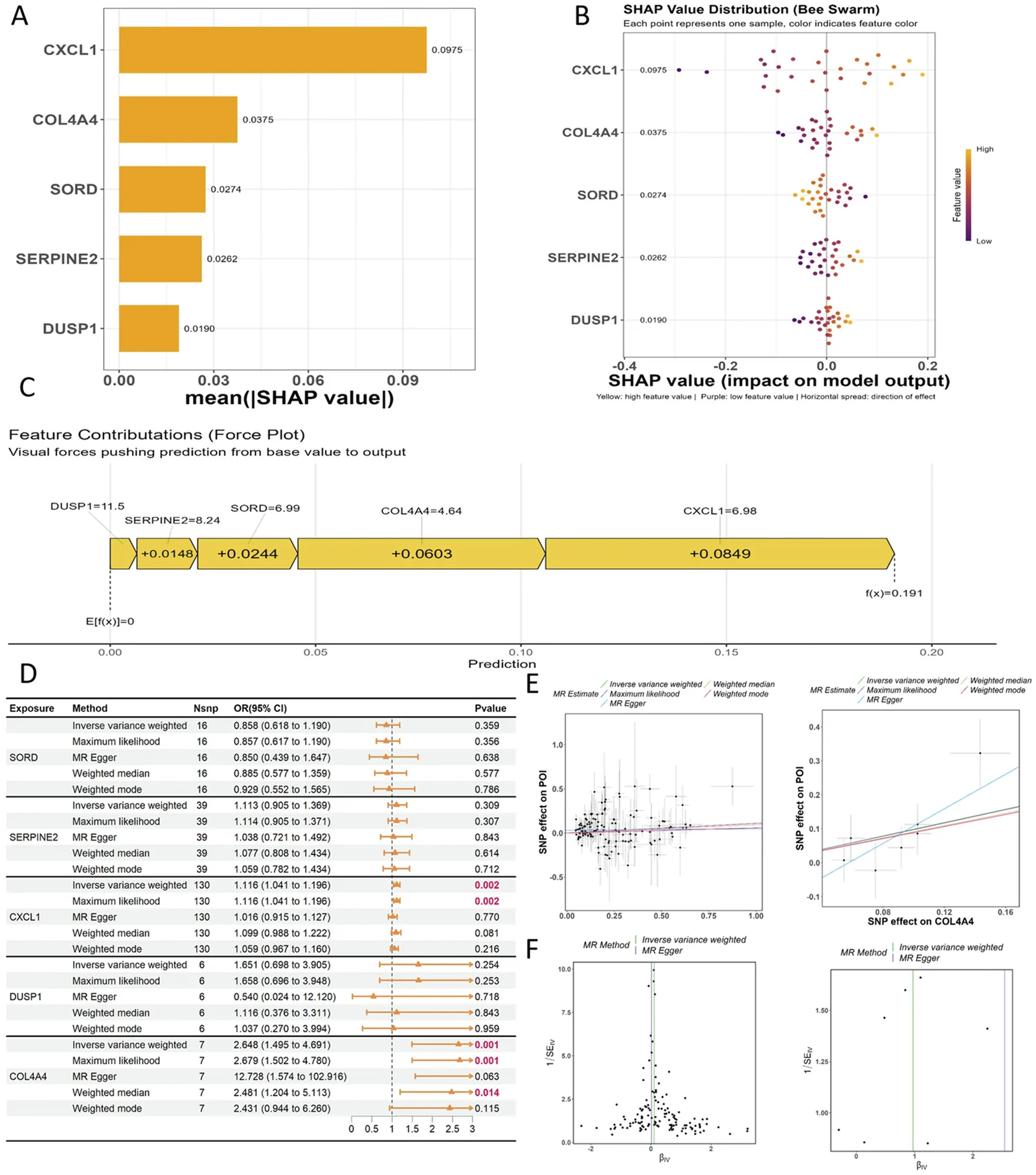
SHAP-based interpretability analysis of the PLS model and MR. **(A)** SHAP bar plot showing overall feature importance based on mean absolute SHAP values. **(B)** SHAP beeswarm plot illustrating the distribution, magnitude, and direction of feature effects across individual samples, with colors indicating feature expression levels. **(C)** SHAP force plot depicting the contributions and directional effects of individual features on the prediction for a representative sample. **(D)** Forest plot showing the genetic associations between genetically predicted gene expression and POI risk. **(E)** Scatter plots illustrating the associations between genetic instruments and POI risk. **(F)** Funnel plots assessing heterogeneity and horizontal pleiotropy.

### 3.7 Mendelian randomization analysis reveals genetic associations of CXCL1 and COL4A4 with POI risk

MR analysis was performed to investigate whether genetically predicted expression levels of CXCL1 and COL4A4 were associated with POI susceptibility. Using POI as a proxy phenotype, five complementary MR approaches consistently demonstrated positive genetic associations between CXCL1/COL4A4 expression and POI risk (Figure 4D). The regression slopes from different MR methods showed similar positive trends, supporting the robustness and consistency of the observed associations (Figure 4E). Funnel plots showed approximately symmetrical distributions of SNP effects around the IVW and MR-Egger regression lines, suggesting no substantial evidence of directional pleiotropy and supporting the reliability of the MR estimates (Figure 4F).

### 3.8 Establishment of the POI rat model induced by CTX

The POI rat model was established via intraperitoneal injection of CTX (Figure 5A). At the end of modeling, both body weight and ovarian weight were significantly lower in the POI group compared to the control group (*P* < 0.01) (Figures 5B–D). Vaginal smears showed that the estrous cycle of the control group rats was normal, while that of the POI group was disordered (Figure 5E). ELISA results showed that serum FSH levels were significantly elevated in the POI group, while E2 and AMH levels were markedly reduced compared to controls (Figures 5F–H). HE staining demonstrated a significant reduction in the number of developing follicles across developmental stages in POI rats, accompanied by a marked increase in atretic follicles and the presence of persistent corpora lutea (Figures 5I,J). The above results indicate impaired ovarian function and reproductive endocrine disorders, suggesting that the CTX-induced POI model was successfully established.

**FIGURE 5:**
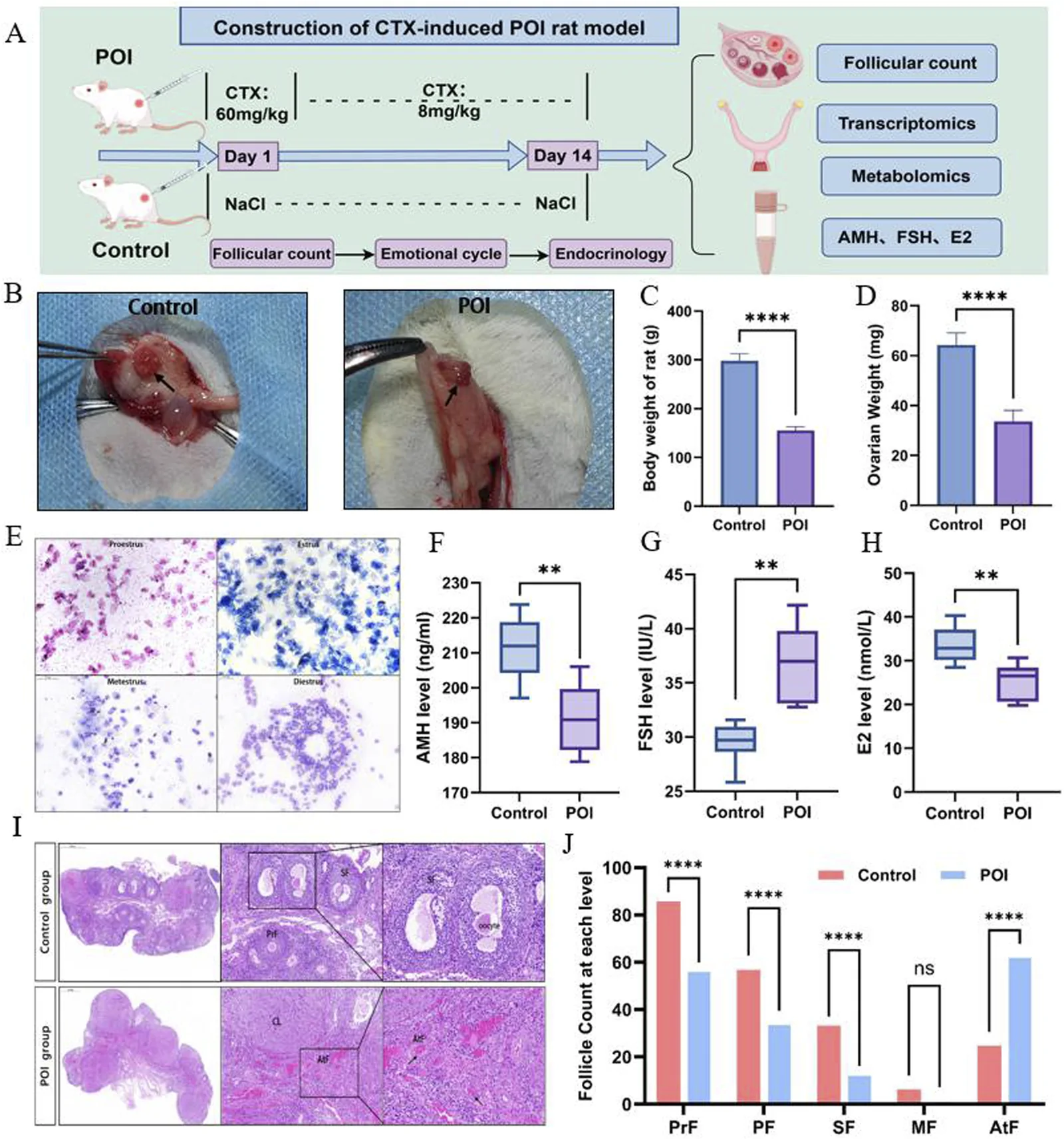
Construction of POI rat model induced by CTX. **(A)** Flowchart for the constructing POI rat model and phenotypic experiments. **(B–D)** The ovarian morphology, body weight and ovarian weight of POI and control group rats. **(E)** Representative images of vaginal smears at different periods. **(F–H)** AMH, FSH and E2 levels in the two groups rats. **(I,J)** HE staining and follicle count of ovaries in the two groups rats.

### 3.9 Transcriptomic characterization of CTX-induced ovarian injury in rats

Using full-length transcriptome sequencing, we systematically characterized the transcriptional landscape of CTX-induced ovarian injury. Boxplot analysis of CPM values demonstrated highly consistent expression distributions across samples, indicating good data quality and inter-sample comparability (Figure 6A). Differential expression analysis identified a total of 2065 DEGs between the POI and control groups, including 1,099 upregulated and 966 downregulated genes (Figure 6B). Hierarchical clustering heatmaps illustrated distinct expression patterns of DEGs across samples (Figure 6C). KEGG pathway enrichment analysis revealed that these DEGs were predominantly involved in pathways related to ovarian steroidogenesis, TNF signaling, steroid biosynthesis, and cytokine–cytokine receptor interaction, highlighting profound disturbances in endocrine regulation and inflammatory signaling in CTX-induced ovarian injury (Figure 6E). GSEA further showed significant positive enrichment of pathways associated with apoptosis, estrogen response late, and fatty acid metabolism in POI samples (NES >1, *P* < 0.05) (Figures 6F–H). In addition, an integrated miRNA–transcription factor–target gene regulatory network was constructed based on the five identified hub targets, providing systems-level regulatory insights into the molecular mechanisms underlying CTX-induced chemotherapy-related ovarian injury (Figure 6D).

**FIGURE 6:**
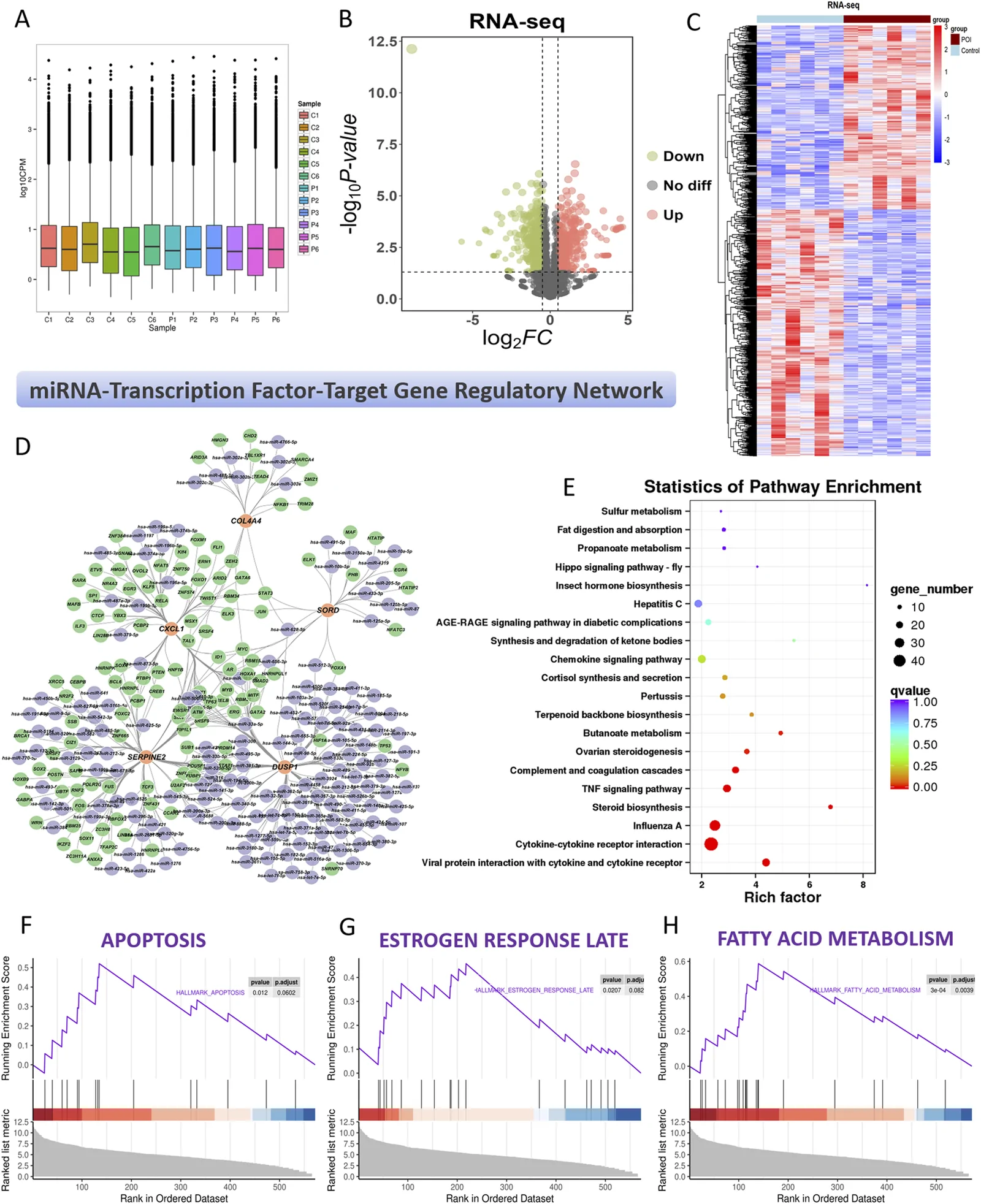
Transcriptomic characteristics of CTX-induced ovarian injury in rats. **(A)** Boxplots of counts per million showing the expression distribution across samples. **(B,C)** Volcano plot and hierarchical clustering heatmap of differentially expressed genes. **(D)** miRNA–transcription factor–target gene regulatory network. **(E)** KEGG pathway enrichment analysis of DEGs. **(F–H)** Gene set enrichment analysis identifying significantly enriched pathways.

### 3.10 Immunohistochemical verification of CXCL1 and COL4A4

IHC analysis demonstrated that CXCL1 protein expression was markedly increased in the ovaries of CTX-induced POI rats compared with control animals. Strong immunoreactive staining was predominantly observed in granulosa cells and stromal compartments, whereas only weak staining was detected in control ovaries. In contrast, COL4A4 expression showed a decreasing trend in POI ovaries compared with controls, although the difference did not reach statistical significance. COL4A4 immunoreactivity was mainly localized in granulosa cells and stromal regions with moderate staining intensity. Notably, the protein expression patterns were largely consistent with the transcriptomic findings, supporting CXCL1 and COL4A4 as potential biomarkers associated with CTX-induced ovarian injury (Figures 7A–C).

**FIGURE 7:**
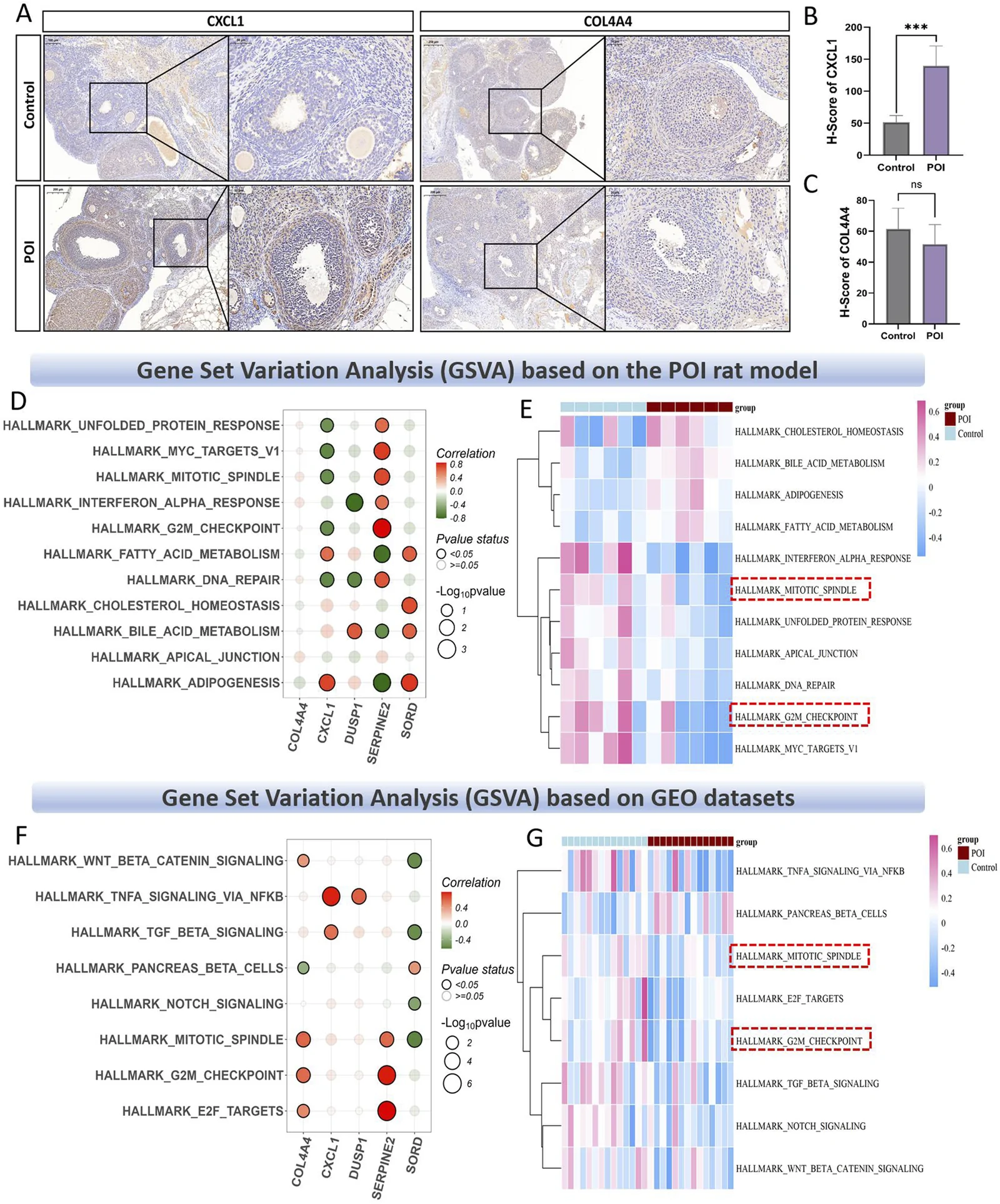
Immunohistochemical and GSVA analyses. **(A–C)** Representative immunohistochemical images and semi-quantitative analysis of CXCL1 and COL4A4 expression in the ovaries of rats from two groups. **(D,E)** Bubble plot of correlations between the expression of five biomarker genes and GSVA pathway scores, and heatmap of differential GSVA scores based on the POI rat ovarian transcriptome. **(F,G)** Corresponding correlation bubble plot and GSVA score heatmap based on the GEO dataset.

### 3.11 GSVA analysis of transcriptomic and GEO datasets

GSVA based on transcriptomic revealed that the POI group exhibited globally negative enrichment scores in cell cycle–and proliferation-related pathways, including Mitotic spindle, G2M checkpoint, MYC targets V1, DNA repair, indicating suppressed mitotic activity and impaired DNA damage response in ovarian tissue. In parallel, multiple metabolic pathways—such as fatty acid metabolism, adipogenesis cholesterol homeostasis, bile acid metabolism—displayed pronounced pathway reprogramming in the POI group, suggesting extensive disruption of lipid metabolism and energy homeostasis following CTX exposure (Figures 7D,E).

Consistently, GSVA of GEO dataset demonstrated widespread downregulation of Mitotic spindle, E2F targets and G2M checkpoint, accompanied by coordinated suppression of TGF-β, NOTCH, and WNT/β-catenin signaling, which are critically involved in cell fate determination, tissue homeostasis, and developmental regulation (Figure 7F). Notably, both datasets converged on the downregulation of Mitotic spindle and G2M checkpoint pathways in POI, highlighting cell-cycle arrest and mitotic dysfunction as central pathological mechanisms underlying chemotherapy-induced POI. Further correlation analysis between the five gene markers and GSVA-enriched pathways indicated that SERPINE2 showed a significant positive correlation with the G2M checkpoint in both the transcriptomic and GEO datasets (Figures 7E,G).

### 3.12 Single-cell transcriptomic analysis identifies ovarian cell populations

To characterize the cellular composition of ovarian tissues and provide additional cellular context for candidate biomarkers, single-cell transcriptomic analysis was performed using a publicly available ovarian dataset. Dimensionality reduction and unsupervised clustering identified 14 distinct cell clusters, which were subsequently annotated into seven major ovarian cell populations based on established marker genes. These included granulosa cells (Fshr, Amh, and Fst), macrophages (Cd68 and Mrc1), endothelial cells (Cd34 and Vwf), epithelial cells (Epcam), luteal cells (Ephx2), stromal cells (Col1a1 and Col1a2), and theca cells (Cyp17a1) (Figures 8A,B). The expression patterns of canonical marker genes across clusters confirmed the reliability of cell-type annotation and demonstrated the heterogeneity of ovarian cellular compartments.

**FIGURE 8:**
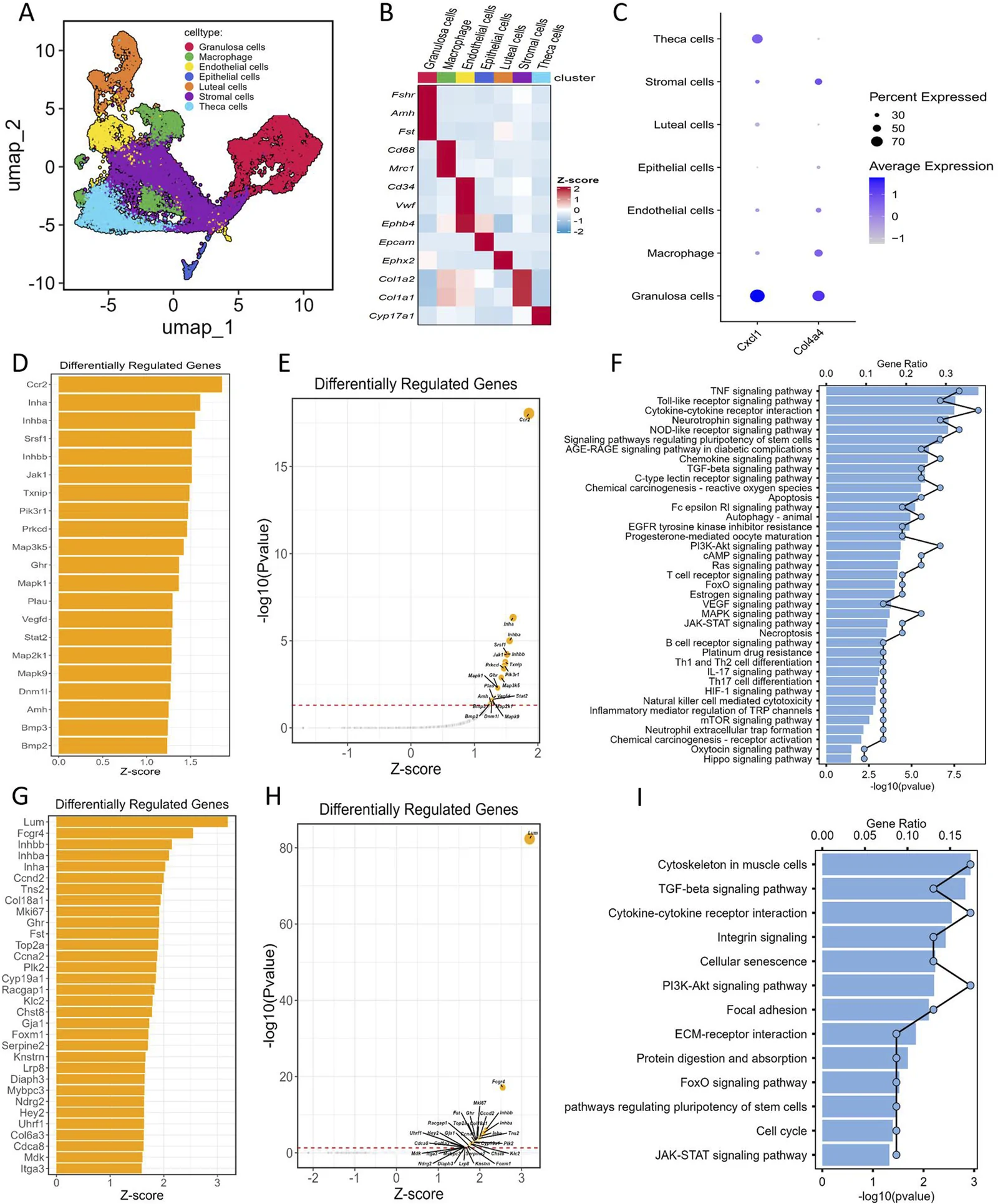
Single-cell-based *in silico* perturbation analysis of CXCL1 and COL4A4. **(A,B)** UMAP visualization of single-cell clusters and heatmap showing the expression patterns of canonical marker genes for cell-type annotation. **(C)** Dot plot depicting the expression distribution of *Cxcl1* and *Col4a4* across different ovarian cell populations. **(D–F)** Ranked distribution of significantly affected genes, differential regulatory gene analysis, and KEGG pathway enrichment analysis following *Cxcl1* virtual perturbation. **(G–I)** Ranked distribution of significantly affected genes, differential regulatory gene analysis, and KEGG pathway enrichment analysis following *Col4a4* virtual perturbation.

### 3.13 Single-cell expression profiling and *in silico* perturbation analysis of *Cxcl1* and *Col4a4*

The cellular distribution of the candidate biomarkers was further investigated at single-cell resolution. *Cxcl1* was predominantly expressed in granulosa cells, with additional expression detected in theca cells. In contrast, *Col4a4* showed an enriched expression pattern in granulosa cells, with detectable expression also observed in stromal populations. In contrast, *Col4a4* showed an enriched expression pattern in granulosa cells, with detectable expression also observed in macrophages and theca cells (Figure 8C). To explore the potential functional consequences associated with these candidate genes, single-cell transcriptome-based *in silico* perturbation analysis was performed in granulosa cells. Virtual perturbation of *Cxcl1* resulted in 21 significantly affected genes, which were mainly enriched in inflammatory and immune-related pathways, including TNF signaling, NF-κB signaling, MAPK signaling, and apoptosis pathways (Figures 8D–F). Similarly, virtual perturbation of *Col4a4* altered 32 significantly affected genes, with functional enrichment highlighting pathways associated with integrin signaling, extracellular matrix (ECM)–receptor interaction, cell adhesion, and cytoskeletal regulation (Figures 8G–I).

### 3.14 Metabolomic characterization of CTX-induced chemotherapy-associated ovarian injury

OPLS-DA score plots showed clear separation between the control and POI groups. Permutation testing indicated good model validity with no apparent overfitting, supporting substantial metabolic alterations associated with CTX-induced POI (Figures 9A,B). Differential analysis identified a total of 162 significantly altered metabolites, including 51 upregulated and 111 downregulated species in the POI group (Figure 9C). And the top 10 upregulated and downregulated DEMs in Figure 9D. Hierarchical clustering analysis revealed distinct abundance patterns of these metabolites across samples (Figure 9E). KEGG pathway enrichment analysis indicated that the DEMs were mainly involved in antifolate resistance, pyrimidine metabolism, NOD-like receptor signaling, vitamin digestion and absorption, ubiquinone and other terpenoid–quinone biosynthesis, and drug metabolism via cytochrome P450, highlighting extensive metabolic reprogramming associated with CTX-induced ovarian injury (Figures 9F,G).

**FIGURE 9:**
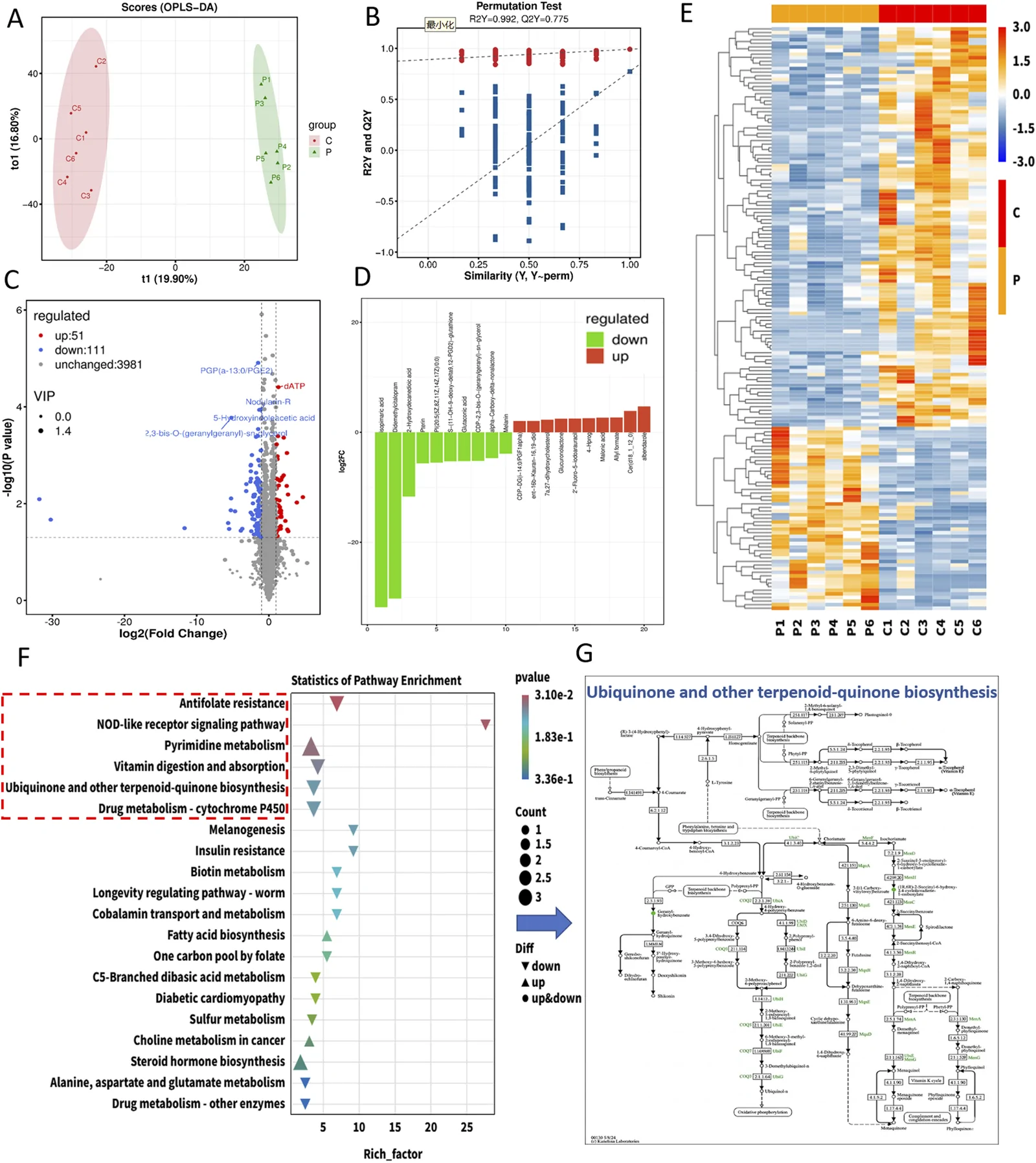
Metabolomics characterized the metabolic changes of CTX-induced ovarian toxicity. **(A,B)** OPLS-DA score plot and corresponding permutation test. **(C,D)** Volcano plot and top 10 upregulated and downregulated differential metabolites. **(E)** Clustered heat map of DEMs. **(F,G)** KEGG pathway enrichment analysis and ubiquinone and other terpenoid-quinone biosynthesis.

## 4 Discussion

This study represents the first integrative framework combining network toxicology, transcriptomics, and metabolomics with machine learning, MR to systematically identify biomarkers and elucidate the molecular mechanisms underlying CTX–induced POI. Using independent training and validation datasets, PLS regression was consistently identified as the optimal diagnostic model, with ROC and calibration analyses demonstrating robust discriminative performance and clinical applicability. SHAP-based interpretability analysis further highlighted CXCL1 and COL4A4 as the most influential contributors to model prediction, implicating their central roles in CTX-induced ovarian injury. Notably, Mendelian randomization analysis supported genetic associations between genetically predicted CXCL1 and COL4A4 expression and POI risk.

CXCL1 is a key chemokine predominantly expressed by neutrophils, macrophages, and epithelial cells, where it plays a central role in inflammatory responses and immune cell recruitment (León et al., 2024). Cisplatin-induced inflammation is driven by a robust CXCL1 response (Ganguly et al., 2024), and upregulation of CXCL1 has been reported as an early indicator of inflammation and immune cell migration in cisplatin-induced ototoxicity (Al Aameri et al., 2023), suggesting that chemotherapy-related tissue injury may share an early chemokine induction–immune cell recruitment–inflammatory amplification cascade. In addition, CXCL1 contributes to cell senescence through regulation of the senescence-associated secretory phenotype (SASP) (Peng et al., 2024). Mechanistically, CTX-induced DNA damage and cellular stress may enhance CXCL1 expression, which in turn binds to its receptor CXCR2 to promote neutrophil infiltration and neutrophil extracellular trap (NET) formation (Pan et al., 2024). This process exacerbates ovarian cell injury and follicular microenvironment disruption through increased reactive oxygen species (ROS) production and apoptosis-related signaling (Tang et al., 2023). COL4A4 encodes the α4 chain of type IV collagen, a fundamental structural component of the basement membrane that is essential for maintaining extracellular matrix (ECM) integrity, cell adhesion, and tissue mechanical stability (Pokidysheva et al., 2025; El-Merhie et al., 2025). Beyond its structural role, type IV collagen actively participates in cell adhesion–mediated signaling. Previous studies have shown that type IV collagen interacts with integrin receptors to regulate cell proliferation, migration, and survival (Öhlund et al., 2013), as well as estrogen receptor expression and function (Novaro et al., 2003). In addition, type IV collagen contributes to ECM remodeling and tissue regeneration through interactions with other extracellular matrix proteins (El-Merhie et al., 2025; Bignon et al., 2011).

Transcriptomic profiling delineated the molecular features of CTX-induced ovarian injury. KEGG enrichment analysis revealed that DEGs were predominantly enriched in *cytokine–cytokine receptor interaction* and *TNF signaling* pathways, consistent with previous reports showing TNF-related and cytokine-mediated signaling as major inflammatory axes in aging regulatory T cells (Zhou et al., 2023). These findings indicate pronounced activation of local inflammatory and immune-related pathways in POI ovaries. Chemotherapeutic agents are known to trigger cytokine release and inflammatory cascades, thereby exacerbating granulosa cell dysfunction and follicular loss (Kawano et al., 2025). Our transcriptomic data provide further support for a critical role of the inflammatory microenvironment in CTX-induced ovarian damage. In addition, significant enrichment of *ovarian steroidogenesis* and *steroid biosynthesis* pathways suggests that CTX profoundly disrupts ovarian endocrine homeostasis and steroid hormone production. GSEA further demonstrated significant activation of *apoptosis* and *estrogen response late* pathways in POI ovaries (NES >1, *P* < 0.05), consistent with our previous transcriptomic findings in peripheral blood from POI patients (Yu et al., 2023a). Notably, CTX-induced granulosa cell apoptosis represents a central pathological driver of follicular depletion and POI (Gu et al., 2025; Chen et al., 2023), while enrichment of the *estrogen response late* pathway likely reflects a compensatory or dysregulated transcriptional response to estrogen deficiency rather than intact estrogen signaling. Together, Single-cell-based *in silico* perturbation analysis suggested that *Cxcl1* and *Col4a4* may be involved in ovarian dysfunction through complementary regulatory programs involving inflammatory responses and extracellular matrix remodeling.

Notably, GSVA analyses of both the ovarian transcriptome and independent GEO datasets consistently identified suppression of the mitotic spindle and G2/M checkpoint pathways in POI. The mitotic spindle is essential for accurate chromosome segregation and successful mitosis (Zhou et al., 2020), whereas the G2/M checkpoint serves as a critical safeguard preventing cell cycle progression in response to DNA damage (Yue et al., 2024). Concurrent downregulation of these pathways indicates that CTX-induced ovarian injury is characterized by cell-cycle arrest, impaired mitotic progression, and dysregulated DNA damage responses. In the ovary, such cell-cycle disturbances can prevent oocytes from completing normal division, thereby accelerating follicular atresia and depletion of the ovarian reserve (Frost et al., 2021). In addition, ovarian granulosa cells are highly proliferative and critically dependent on precise cell-cycle regulation; therefore, suppression of the G2/M checkpoint may rapidly trigger apoptotic signaling and disrupt the follicular microenvironment (Yan et al., 2019). Base on GSVA findings, our results suggest that CTX-induced ovarian toxicity may be driven by a coordinated “DNA damage–cell-cycle arrest–inflammatory and immune amplification” cascade, providing a mechanistic explanation for the ovary’s pronounced sensitivity as a primary target organ of CTX toxicity.

Untargeted metabolomics is a powerful approach to characterize drug-induced metabolic perturbations, which often provide mechanistic insights into drug toxicity (Cuykx et al., 2019). KEGG enrichment analysis of DEMs revealed pronounced metabolic reprogramming in CTX-induced POI ovaries. Downregulation of the *antifolate resistance* pathway indicates impaired folate-dependent one-carbon metabolism and nucleotide biosynthesis (Raz et al., 2016), which may limit DNA synthesis and repair capacity and reinforce cell cycle arrest and apoptosis. This metabolic deficiency is highly concordant with transcriptomic suppression of *DNA repair*, *G2/M checkpoint*, and *mitotic spindle* pathways, supporting a coordinated disruption of genome maintenance. Concurrently, suppression of *ubiquinone and other terpenoid–quinone biosynthesis* suggests reduced coenzyme Q10 availability, implicating mitochondrial dysfunction, impaired oxidative phosphorylation, and compromised redox homeostasis (Hidalgo-Gutiérrez et al., 2021; Yu et al., 2023b). Mitochondria affect the function of ovarian granulosa cells and lead to ovarian aging by regulating cellular energy metabolism, intensifying oxidative stress and mitochondrial DNA (mtDNA) damage (Zhang et al., 2025). In addition, *drug metabolism–cytochrome P450* represents a central pathway governing xenobiotic activation and detoxification (Rodriguez-Antona et al., 2010). Downregulation of *drug metabolism–cytochrome P450* pathways implies diminished ovarian detoxification capacity, potentially prolonging local exposure to CTX and its reactive metabolites (Rodriguez-Antona and Ingelman-Sundberg, 2006; Helsby et al., 2019). Given the dependence of CYP450 enzymatic activity on mitochondrial energy metabolism (Child et al., 2020), CTX-induced mitochondrial dysfunction may further suppress drug metabolism, establishing a self-amplifying cycle that exacerbates DNA damage and ovarian toxicity.

Several limitations should be considered. First, the CTX regimen used in the rat model does not fully reproduce clinical dosing schedules, combination therapies, or supportive care, and the timing and severity of ovarian injury may therefore differ from those observed in patients. Studies using clinically relevant exposure designs and, where possible, patient-derived samples will be needed to strengthen translational applicability. Second, CXCL1 and COL4A4 were prioritized through machine learning, Mendelian randomization, transcriptomic analysis, single-cell-based in silico perturbation analysis, and immunohistochemical validation, but their functional roles in CTX-induced ovarian injury remain to be established. Future studies should examine CXCL1 and COL4A4 knockdown and overexpression in granulosa cells and animal models, together with rescue experiments, to determine whether these candidates directly regulate follicular injury and ovarian function. Third, the transcriptomic and metabolomic findings currently demonstrate concurrent immune-inflammatory activation, impaired DNA damage responses, and metabolic disturbances rather than a proven causal cell cycle, DNA damage repair, and metabolic alterations. Matched gene–metabolite analyses and targeted perturbation experiments will be required to establish pathway directionality and determine whether modulation of one omponent alters the other molecular processes and improves ovarian phenotypes.

## 5 Conclusion

In conclusion, this study applied an integrated network toxicology and multi-omics strategy combined with machine learning and genetic analyses to characterize candidate biomarkers and molecular alterations associated with CTX-induced ovarian toxicity. The PLS model showed robust predictive performance, while CXCL1 and COL4A4 were prioritized as key contributors to model discrimination. Transcriptomic analyses revealed suppression of cell cycle–related pathways, including the G2/M checkpoint and mitotic spindle, together with activation of inflammatory and apoptotic signaling, consistent with disrupted DNA damage responses and impaired ovarian cellular homeostasis. Metabolomic analyses further identified coordinated alterations in folate metabolism, ubiquinone biosynthesis, and cytochrome P450–mediated drug metabolism, suggesting disturbances in mitochondrial function, nucleotide metabolism, and redox balance. In addition, single-cell-based *in silico* perturbation analysis suggested that CXCL1 and COL4A4 may contribute to ovarian dysfunction through regulation of inflammatory responses and extracellular matrix remodeling. Collectively, these findings indicate that CTX-induced ovarian injury involves interconnected immune-inflammatory, DNA damage response, and metabolic alterations, providing potential molecular insights into chemotherapy-associated ovarian dysfunction and fertility preservation (Figure 10).

**FIGURE 10:**
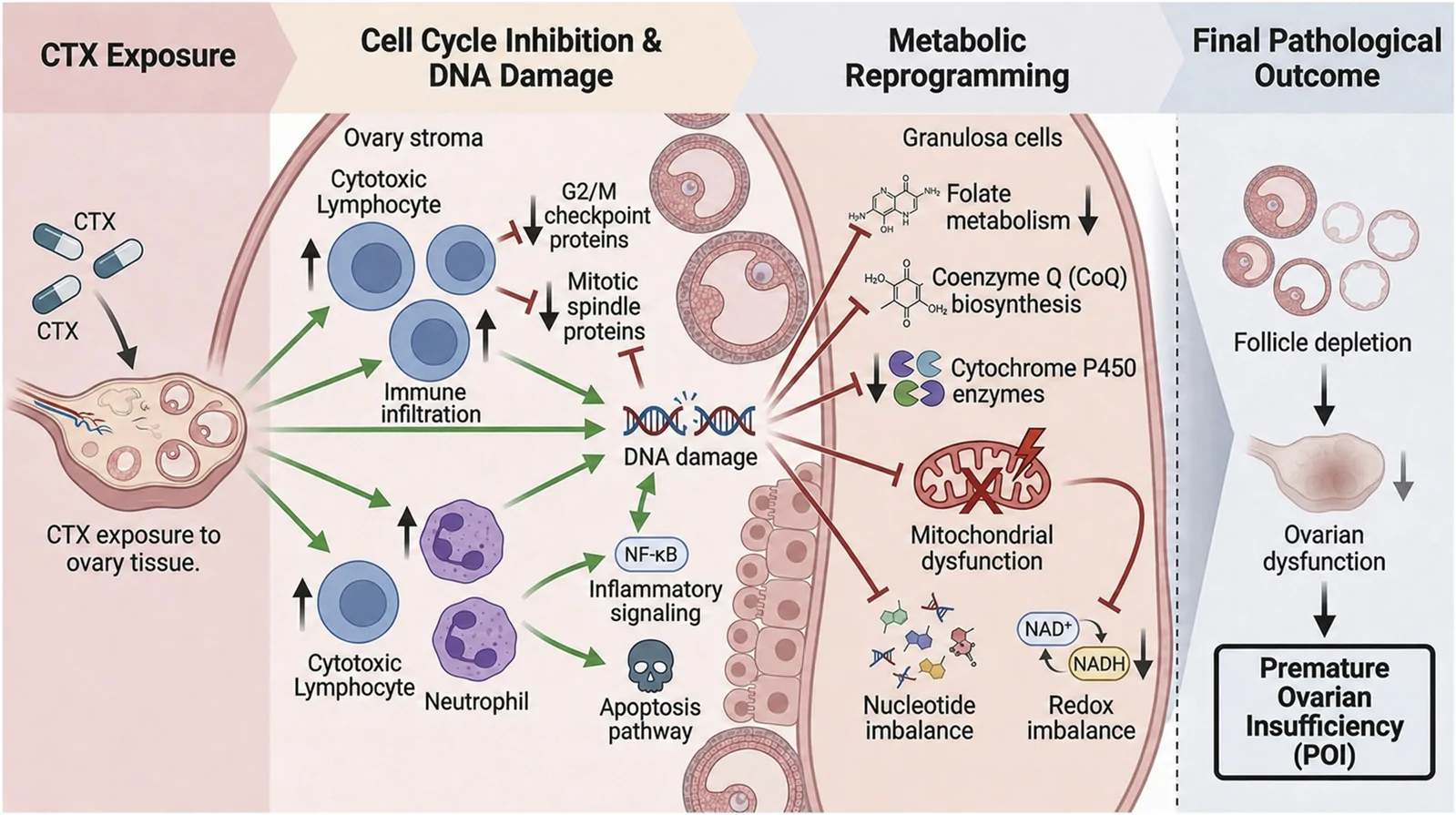
Clarify the mechanism of CTX-induced ovarian toxicity based on transcriptomics and metabolomics.

## Data Availability

The sequencing data presented in this study are deposited in the Genome Sequence Archive (GSA) of the National Genomics Data Center (NGDC), accession number CRA046425. Additional supporting data are provided in the Supplementary Materials.
